# Pilot comparison of outcome measures across chemical and surgical experimental models of chronic osteoarthritis in the rat (*Rattus norvegicus*)

**DOI:** 10.1371/journal.pone.0277943

**Published:** 2022-11-21

**Authors:** Morika D. Williams, Rachel C. Meyers, Lauryn A. Braxton, Brian Diekman, B. Duncan X. Lascelles

**Affiliations:** 1 Division of Comparative Medicine, Department of Pathology and Laboratory Medicine, University of North Carolina at Chapel Hill, Chapel Hill, North Carolina, United States of America; 2 Translational Research in Pain Program, North Carolina State University, Raleigh, North Carolina, United States of America; 3 Comparative Pain Research and Education Center, North Carolina State University, Raleigh, North Carolina, United States of America; 4 Comparative Medicine Institute, North Carolina State University, Raleigh, North Carolina, United States of America; 5 Division of Rheumatology, Allergy, and Immunology, Thurston Arthritis Center, University of North Carolina at Chapel Hill, Chapel Hill, North Carolina, United States of America; 6 Department of Clinical Sciences, College of Veterinary Medicine, North Carolina State University, Raleigh, North Carolina, United States of America; 7 Department of Molecular and Structural Biochemistry, North Carolina State University, Raleigh, North Carolina, United States of America; 8 Joint Department of Biomedical Engineering, University of North Carolina at Chapel Hill and North Carolina State University, Raleigh, North Carolina, United States of America; 9 Center for Translational Pain Medicine, Duke University, Durham, North Carolina, United States of America; University of Vermont College of Medicine, UNITED STATES

## Abstract

Relatively little work has evaluated both the disease of osteoarthritis (OA) and clinically-relevant pain outcome measures across different OA models in rats. The objective of this study was to compare sensitivity, pain, and histological disease severity across chemical and surgical models of OA in the rat. Stifle OA was induced in Sprague–Dawley rats via intraarticular injection of monoiodoacetate (MIA) or surgical transection of anterior cruciate ligament and/or destabilization of medial meniscus (ACL+DMM or DMM alone). Reflexive (e.g., mechanical and thermal stimuli) measures of sensitivity and non-reflexive assays (e.g., lameness, static hindlimb weight-bearing asymmetry, dynamic gait analysis) of pain were measured over time. Joint degeneration was assessed histologically. Six-weeks post OA-induction, the ACL+DMM animals had significantly greater visually observed lameness than MIA animals; however, both ACL+DMM and MIA animals showed equal pain as measured by limb use during ambulation and standing. The MIA animals showed increased thermal, but not mechanical, sensitivity compared to ACL+DMM animals. Joint degeneration was significantly more severe in the MIA model at 6 weeks. Our pilot data suggest both the ACL+DMM and MIA models are equal in terms of clinically relevant pain behaviors, but the MIA model is associated with more severe histological changes over time potentially making it more suitable for screening disease modifying agents. Future work should further characterize each model in terms of complex pain behaviors and biochemical, molecular, and imaging analysis of the sensory system and joint tissues, which will allow for more informed decisions associated with model selection and investigative outcomes.

## Introduction

Osteoarthritis (OA) is a lifelong degenerative disease affecting all components of the joint and can be associated with pain. The prevalence of OA increases with age, with approximately 33.6% of adults 65 and older having OA [1]. However, other etiological factors play a role in the development of OA, including sex, obesity, genetic predilection, and a history of trauma and occupational injury. However, the relationship between measurable disease and pain is complex and nonlinear [1]. Joint pain and arthritis account for 58% of all painful conditions, significantly contributing to the economic burden of persistent pain [2]. Persistent joint pain can interfere with a person’s quality of life by impairing mobility, the ability to perform activities of daily living, and creating anxiety, cognitive dysfunction, and impaired mood. Current options for pain management in OA patients is considered suboptimal [3], and translational pain research is not producing novel, effective, and safe therapeutic options [4]. There has been increased discussion about how to optimize translational pain research [5–7].

Induced rodent models of OA are critically important to the discovery of novel therapeutics, and they are used to assess both putative disease-modifying agents and putative analgesics. To date, there is not an agreement on one gold standard experimental rodent model of OA. Both surgical and chemical models of OA in rodents have shown value in studying disease pathways, mechanisms, and treatment targets [5]. The intraarticular injection of monoiodoacetate (MIA) is a well-established OA model used for pain behavior research to study alterations in sensory thresholds, functional impairment in limb use, cartilage degeneration, and novel therapies [8–10]. In terms of cartilage degeneration, the MIA model produces rapid lesions, which does not represent the chronic progression of human OA [11]. Surgical models, such as the destabilization of the medial meniscus (DMM), cause slow, progressive, and site-specific articular cartilage degeneration, osteophytes, and subchondral bone defects in the medial tibial plateaus of the stifle [12, 13], more representative of human OA. Despite numerous studies employing rodent OA models, none have compared different models in rats across clinical alterations (e.g., reflexive, non-reflexive) and histological characteristics.

The objective of this pilot study was to compare the clinical pain-associated alterations and histological severity across commonly used or recently established chemical (i.e., MIA) and surgical (i.e., DMM model, combined DMM and anterior cruciate ligament (ACL) transection) models of OA in the rat. We hypothesized the surgical OA models would have greater pain-associated changes and more localized, slowly progressive, and less severe OA lesions than the chemical OA model. This study may be beneficial in guiding OA model selection.

## Materials and methods

### Animals

Sprague Dawley rats (n = 50; 8–10 weeks old; 278–373 g; *Rattus norvegicus*; SAS Crl:SD; Charles River, Kingston, NY) kept under standard environmentally controlled conditions were used for this study. Animals were randomized within experiment and assigned to injury groups based on availability of animals (Table 1). In this pilot work, more females were used based on the understanding that females are more likely to present with more severe clinical signs of post-operative pain [14]. All animals were pair-housed and clinically healthy prior to study. All experiments were approved by the North Carolina State University Institutional Animal Care and Use Committee (Protocol Number: 16–185) and conducted according to the National Institutes of Health Guide for the Care and Use of Laboratory Animals within AAALAC International-accredited facilities.

**Table 1:** Experimental groups and distribution of male and female animals.

Model	N	Males	Females
**ACL+DMM**	6	0	6
**MIA**	22	9	13
**Saline**	14	7	7
**DMM**	8	4	4
**Intact Control**	6	0	6

### Induction of OA models

For all experimental models described below, rats were anesthetized using inhalation anesthesia (O2: 1.5–2 L/min; Isoflurane: 1.5–2.5%) allowing quick recovery. For chemical induction of OA, a single percutaneous intraarticular injection of 2 mg per rat of MIA (Sigma-Aldrich, St. Louis, MO, USA) or saline vehicle was administered through the infrapatellar ligament of the left stifle joint (n = 22 and 14, respectively) as previously described [15]. This dose was chosen to model severe OA with associated pain, while lower doses (0.03–0.1 mg) produce subtle OA with no associated pain [15, 16]. MIA was dissolved in physiological saline and administered in 25 μL using a 0.3mL 30-gauge 0.5-inch needle insulin syringe. Saline-injected rats (S) received equal volumes. The ACL+DMM (n = 6) and DMM (n = 8) surgical OA models were preemptively administered buprenorphine (0.01 mg/kg, SC) and induced in the left stifle joint as previously described [17]. The skin on the medial aspect of the left stifle was shaved, disinfected, incised together with the underlying muscles, followed by the transection of the ACL and/or the meniscotibial ligament of the medial meniscus, and the skin was closed using absorbable sutures. A single experimenter (MDW) induced OA in all animals. The right contralateral stifle joint was used as a histologic intact control (n = 6). The MIA and ACL+DMM groups were tested at pre-determined intervals for clinical metrics but all three OA groups and the intact controls were used for histology.

### Subjective assessments of overall health

Body weight was recorded weekly for the duration of the study. Changes in body weight from baseline were calculated as an indirect assessment of health status and appetite.

### Behavioral analysis

Behavioral analysis included visual evaluation of lameness, mechanical and thermal reflexive sensitivity, and movement-induced and spontaneous limb use measures. Animals were habituated to the behavioral testing suite for at least 30 minutes before testing. Baseline readings for all tests were established a week prior to OA-induction, then at least weekly over 6-week period. Testing order was randomized by sex at each time point. Experimenters (RCM and LAB) were blinded to experimental groups during assessments performed.

### Subjective assessments of lameness

Lameness assessment was conducted prior to manipulation or testing at cage-side level, while animals were in home cage. Experimenters evaluated the animals’ willingness to ambulate, stance during rearing, and position of hindlimbs at rest. Lameness was scored visually using a 3-point scale [0 = normal, equal weight-bearing on both hindpaws; 1 = abnormal weight-bearing, reduced paw pressure and limb use, paw curled with only some parts of hind paw touching floor; 2 = non-weight-bearing, paw completely elevated]. Evaluations were made by individuals trained to observe lameness in higher order animals, and observations were made over a 10-minute period of observation.

### Reflexive measures of sensitivity

Mechanical stimulus testing was performed before thermal stimulus testing with at least a 15-minute rest period between modalities. Rats were placed individually in Plexiglas chambers on an elevated mesh-grid platform. Mechanical sensitivity was measured by an Electronic von Frey Anesthesiometer (IITC Life Science, Woodland Hills, CA) applied to the midplantar surface of hindpaws. The mean of 5 trials per hindpaw was recorded as the paw withdrawal threshold (PWT).

After a rest period, rats were placed individually in Plexiglas chambers on an elevated room temperature glass platform. The Plantar Test (Hargreaves Apparatus; Ugo Basile, Varese, Italy) was employed to measure thermal sensitivity. An infrared laser beam was placed under the mid-plantar surface of the hindpaw, with a 40-s cut-off time to prevent tissue injury. The mean of 5 trials per hindpaw was recorded as the paw withdrawal latency (PWL).

### Limb use measures

Movement-induced nociception was evaluated using the C*atWalk* (CW; Noldus Informational Technology, Wageningen, Netherlands), an automated gait analysis apparatus, as previously described [18]. Rats traverse an enclosed walkway with a glass floor in a darkened room. A florescent bulb produces internally reflected light within the glass plate, which only scatters at points where a paw touches the glass, producing a bright illumination of the contact area. Stance phase of limb (s), print length of paw (cm), print area of paw (cm^2^), swing phase of limb (s), and time to max paw-floor contact (%) were the selected outcome measures. These were calculated as means across 3 runs, for ipsilateral (left) hindpaw of each rat at every time point.

Spontaneous pain was assessed using the Static Horizontal Incapacitance Meter (SHIM) with animals in normal standing position as we have previously described [19]. Rats were tested 3 consecutive times, with a scan rate of 10 scans per second over 5 seconds. The means across 3 trials were calculated for each hindlimb of each rat at each time point. Percentages of the total bodyweight placed on independent hindlimbs were calculated [19].

### Histological analysis

The stifle joints were harvested at 6 (MIA, ACL+DMM, Intact Controls) and 14 weeks (DMM) after OA-induction. Stifle joints were fixed in 4% paraformaldehyde for 7–10 days, decalcified in 10% formic acid for 48 hours, and then embedded in paraffin wax in frontal orientation. Serial sections were obtained at 200 μm steps (5 μm thick). Sections were excluded if there was poor orientation. Arthritic changes to the medial tibial plateau were quantified from three Safranin-O stained sections using the modified Mankin scoring system [20] and two OARSI scores established by Pritzker [21] and Gerwin scores [22]. Modified Mankin scores evaluated surface structure (0–6), chondrocyte abnormalities (0–3), proteoglycan content (0–4), and tidemark change (0–1), for a maximum possible score of 14 [20]. Pritzker scores evaluated surface and cartilage integrity (grade, 0–6) and extent of joint involvement (stage, 0–4), for a maximum score of 24 [21]. Gerwin scores evaluated width of cartilage matrix loss at surface, mid, and tidemark zones (μm); total width of tibial cartilage degeneration (μm); total width of significant tibial cartilage degeneration (μm); tibial cartilage degeneration score at the medial, center, and inner zone (0-5/zone), for a maximum score of 15; and osteophyte score (0–4) [22].

### Statistical analysis

Statistical analysis was performed using the software program JMP 14 (SAS Institute, Cary, NC, USA). The Wilcoxon Rank Sums test or Steel-Dwass method were used to analyze the difference between means of two or more independent groups, respectively, and to analyze multiple comparisons. We used this approach rather than modeling in order to be more conservative about the statistical conclusions. All assessments were evaluated as independent measures. In all cases, P < 0.05 was considered significant. Data were expressed as mean ± SEM.

## Results

### Clinical evaluation

Animals recovered uneventfully after all OA-induction procedures. All groups gradually gained weight over time with no differences detected between groups, despite differences in measurable indicators of pain.

### Subjective lameness

**Fig 1:**
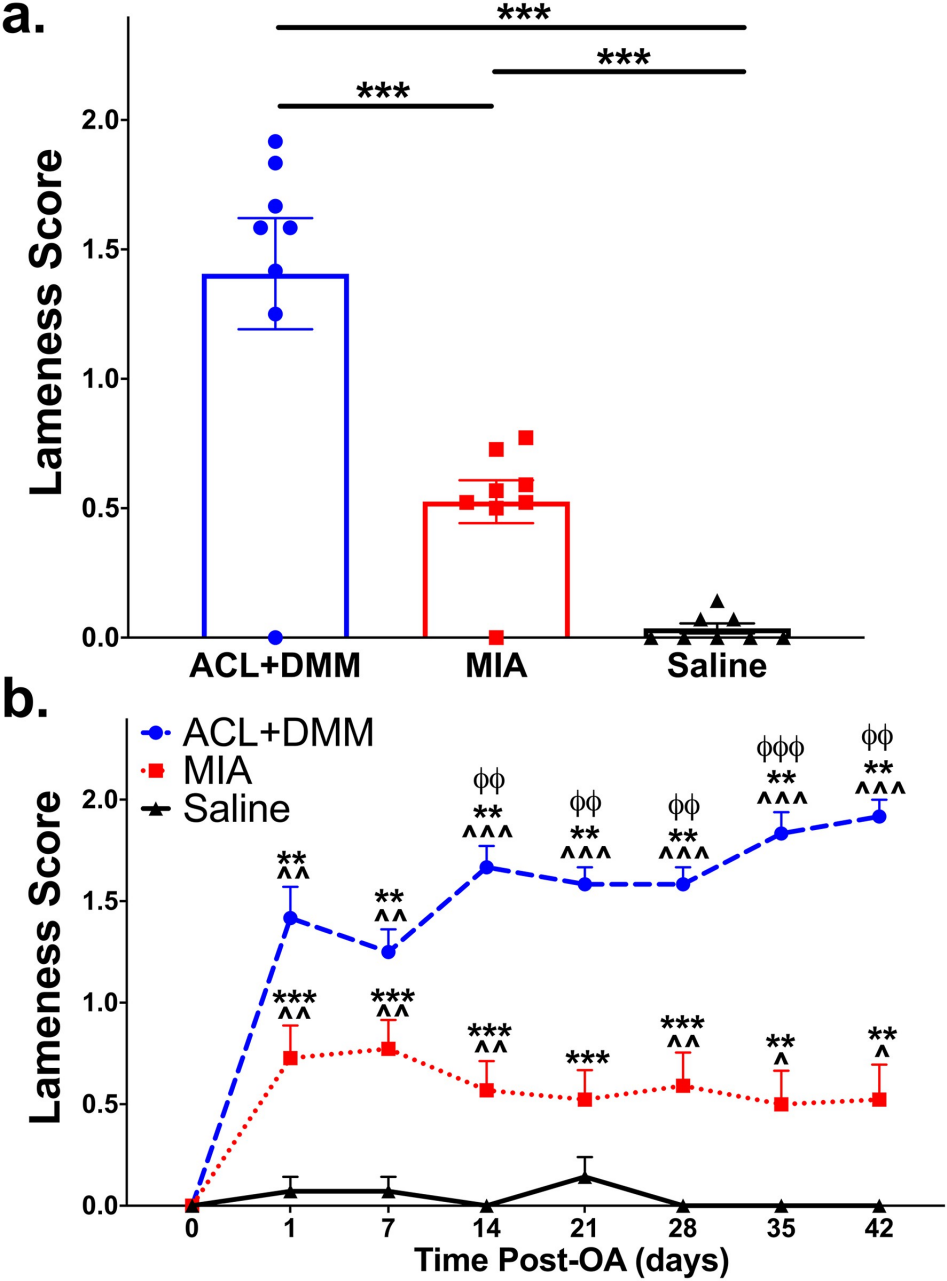
Comparison of lameness scores across OA models. (a) Overall lameness scores. The mean lameness score at each time point is represented by a single data point, with the bar plot showing the mean score across 8 time points for each group, and these were averaged to calculate an overall lameness score for each group. *p<0.05, **p<0.01, ***p<0.001 shows comparisons between individual experimental groups. (b) Lameness scores at each of the 8 time points post-OA induction (x-axis). Groups represented as: ACL+DMM (n = 6), blue (circle); MIA (n = 22), red (square); Saline control (n = 14), black (triangle), with symbol representing group mean at each time point. Data are expressed as the mean ± SEM. *p<0.05, **p<0.01, ***p<0.001 compared to baseline; ^p<0.05, ^^p<0.01, ^^^p<0.001 compared to saline; ^ϕ^p<0.05, ^ϕϕ^p<0.01, ^ϕϕϕ^p<0.001 for ACL+DMM vs MIA.

Overall, both the MIA and ACL+DMM groups appeared visually lame for the 6-week study duration and were significantly more lame than saline controls. Significantly greater lameness was seen in ACL+DMM rats compared to the MIA group. No lameness was seen in saline control rats. A group, by time interaction, was detected in both OA models, but ACL+DMM lameness scores were persistently higher than MIA scores for the duration of the study, and significantly so from D14 to D42 (Fig 1).

### Reflexive responses after OA induction

#### Mechanical sensitivity

Overall, the ipsilateral hindlimb was significantly more sensitive than the contralateral hindlimb following OA-induction. There were no overall group effects detected in the change from baseline in PWT for the ipsilateral hindlimb across models (S1a Fig). Plots of PWT over time showed variable decreases below baseline in all groups, most frequently in the ACL+DMM group, however there were no differences between groups (S1b Fig). Peak mechanical sensitivity was observed on day 8 in MIA group and peak mechanical sensitivity was observed on day 15 the ACL + DMM group.

#### Thermal sensitivity

There was an overall group by PWL interaction detected following OA-induction, with an overall trend towards thermal hypersensitivity in MIA model compared to saline (S1c Fig). Animals in the MIA group slowly became more sensitive to thermal stimuli on their ipsilateral hindlimb by D15, then slowly returned to baseline by D36 (S1d Fig). Changes in thermal sensitivity were not detected in the ACL+DMM group nor saline controls.

These data suggest MIA is associated with a period of thermal sensitivity, but not mechanical sensitivity, suggesting differential effects of the OA model on sensory systems.

### Effect of OA-induced changes on dynamic limb use and static hindlimb weight-bearing

#### Dynamic gait analysis

**Fig 2:**
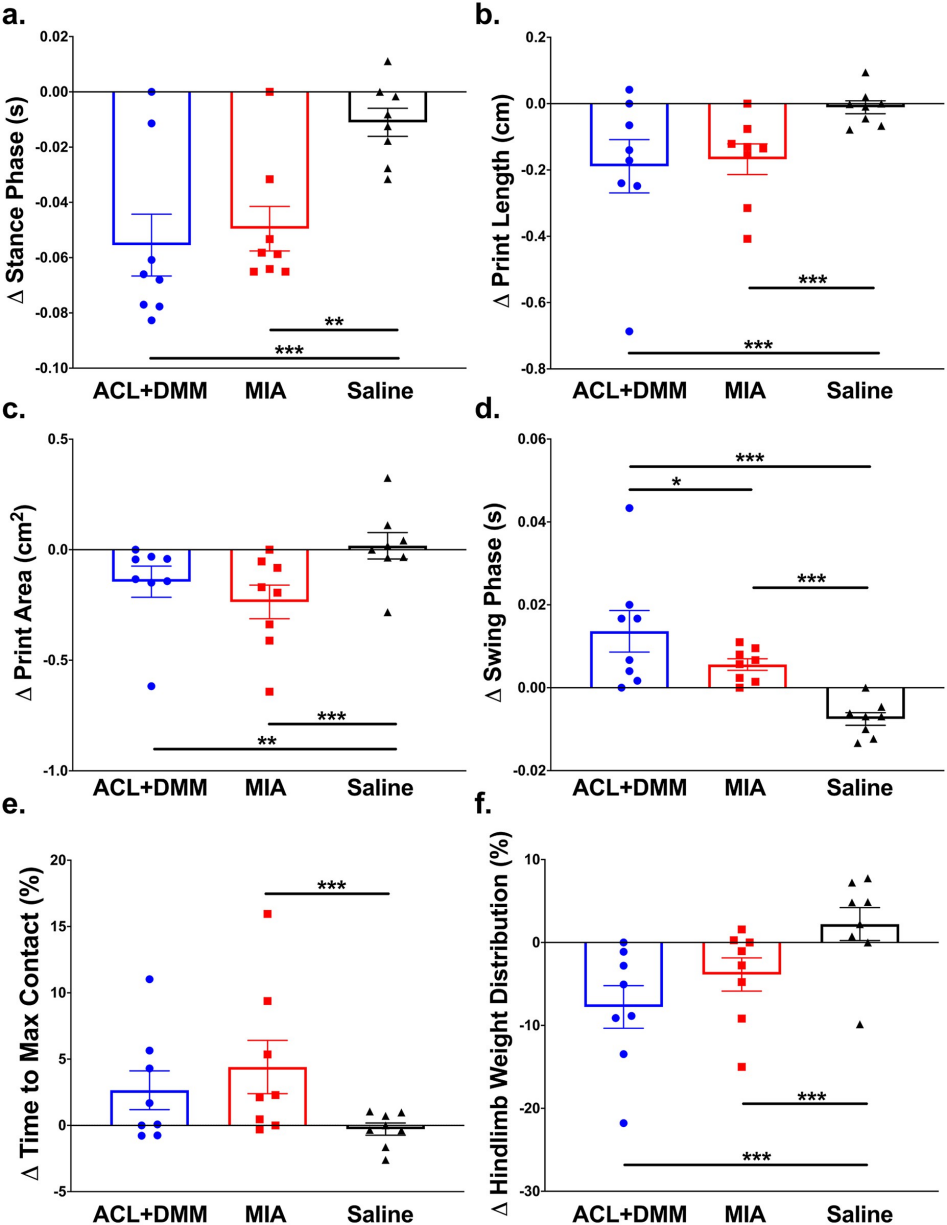
Changes on dynamic and static limb use of ipsilateral hindlimb following the induction of OA. Bar plots show the mean score across 8 time points for each group, which were averaged to calculate an overall (a-e) dynamic gait and (f) static weight distribution on ipsilateral hindlimb following OA-induction shown as overall change for (a) stance phase, (b) print length, (c) print area, (d) swing phase, (e) time to max paw-floor contact, and (f) weight distribution. Groups represented as: ACL+DMM (n = 6), blue (circle); MIA (n = 22), red (square); Saline control (n = 14), black (triangle), with each symbol representing group mean at each time point. Data are expressed as the mean ± SEM. *p<0.05, **p<0.01, ***p<0.001 shows comparisons between individual experimental groups.

Both OA models negatively impacted dynamic limb use when compared to saline controls. The ipsilateral hindlimb of ACL+DMM and MIA groups showed significant overall reductions in stance phase, print length, and print area, as well as increases in swing phase compared to controls across all 8 time points for each group (Fig 2a–2d). Across gait analysis parameters, there were acute effects detected in both OA groups (S2a–S2e Fig). The MIA group had significant increases in time to max paw-floor contact at 1 and 7 days, unlike the ACL+DMM rats.

For stance phase, both OA groups followed a similar pattern where there was a reduction in stance phase from D1 to D7 and D21 to D42 with a temporary return in between (D14); the reduction was slightly greater in the ACL+DMM animal (S2a Fig). The pattern of decreased print length was similar between both OA groups, with an acute reduction at D1, which returned to baseline by D14; the reduction was greater in the ACL+DMM group(S2b Fig). An acute reduction in print area was detected across all groups at D1 with an immediate return to baseline at D7 except in the MIA group, which was significantly lower than baseline at D1, D7, D14, D28, and D35 (S2c Fig). Swing phase acutely increased in both OA groups but significantly more so in the ACL+DMM group, which remained above baseline for the duration of study (S2d Fig). Unlike other gait parameters, the MIA group had a more pronounced increase in time to max paw-floor contact by D1 with a gradual reduction but remained higher than baseline for duration of study, while the ACL+DMM group had an acute peak in max paw floor contract at D1 and remained above baseline. Overall, the two OA groups showed similar changes over time across all dynamic limb use parameters. For all parameters, there were minimal changes over time in the saline control group.

#### Static weight-bearing asymmetry

Chemical and surgical induction of OA resulted in significant weight-bearing asymmetry with reductions in ipsilateral hindlimb weight-bearing after OA-induction (Fig 2f). Both OA groups had an acute reduction in weight-bearing on the ipsilateral hindlimb by D2 with a gradual increase towards baseline by D14; however, weight-bearing slightly decreased again in the ACL+DMM group, but by D42, values were back to baseline and there were no differences between groups (S2f Fig).

### Assessment of stifle joint degeneration by OA induction

**Fig 3:**
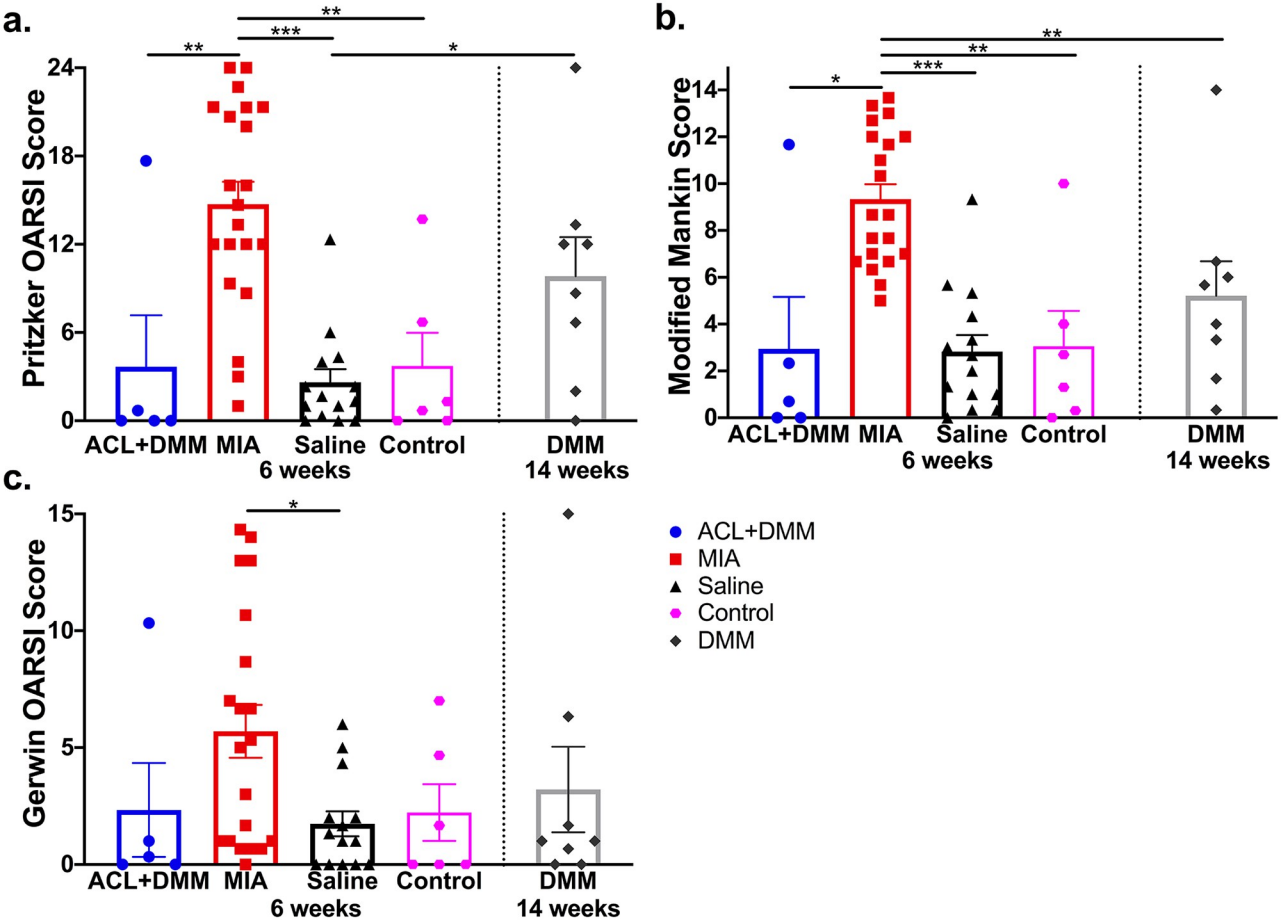
Histological scoring of cartilage damage 6 or 14 weeks following OA induction. Graphical representation of histological grading of ipsilateral medial tibial plateaus at 6 weeks post-OA induction for ACL+DMM, MIA, saline control, and intact control rats and 14 weeks post-OA induction for DMM rats: (a) Pritzker OARSI scores, (b) Modified Mankin score; and (c) Gerwin OARSI scores. Groups represented as: ACL+DMM (n = 5), blue (circle); MIA (n = 21), red (square); Saline control (n = 14), black (triangle); Intact control (n = 6), magenta (octagon); DMM (n = 8), grey (diamond), with each symbol representing an individual animal. Data are expressed as the mean ± SEM. *p<0.05, **p<0.01, ***p<0.001 for group comparisons.

**Fig 4:**
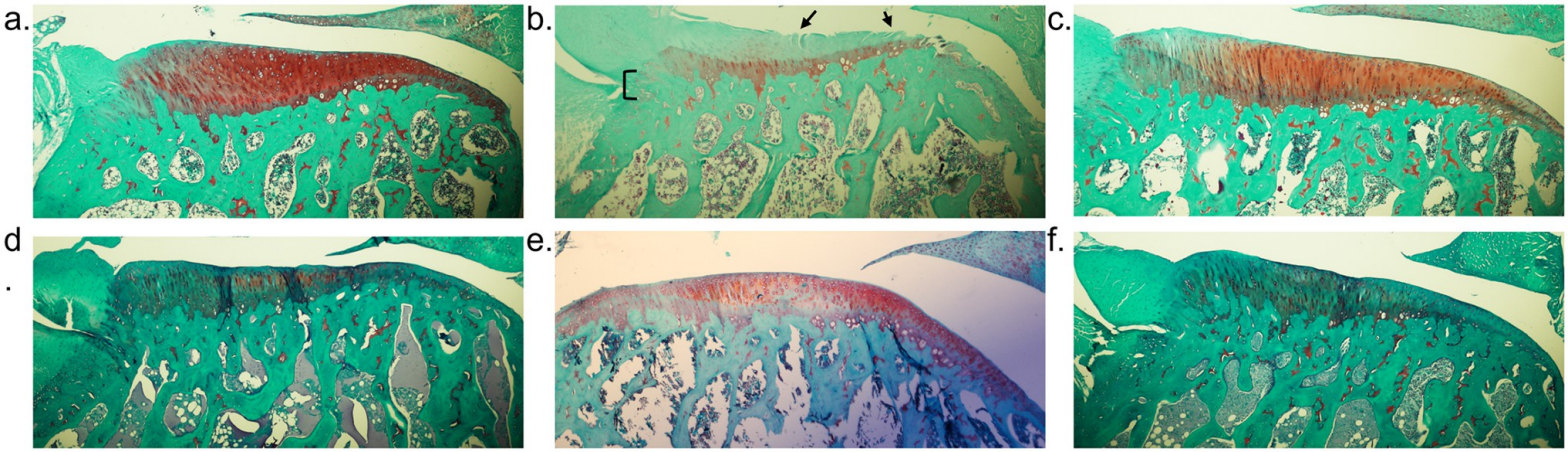
Safranin-O staining of cartilage from medial tibia. Representative histologic images of the femorotibial joint, focusing on the medial side of the tibial surface. (a) saline controls 6 weeks after intraarticular injection, (b) MIA model 6 weeks after intraarticular injection, (c) intact control 6 weeks after anesthesia, (d) ACL+DMM model 6 weeks post-surgical induction, (e) intact control 14 weeks after anesthesia, and (f) DMM model 14 weeks post-surgical procedure. For panel (b) the loss of Safranin-O staining is observed up until the tidemark. Ulceration of articular cartilage (arrow) and extensive bone remodeling (bracket) are observed.

Significant histological evidence of stifle joint degeneration was seen in the ipsilateral hindlimb 43 days post-OA induction in the MIA model when compared to all other groups. These histopathological OA lesions were greater in the MIA model based on modified Mankin, Pritzker, and Gerwin scoring systems (Fig 3). When compared to the medial tibial plateaus, the lateral tibial plateaus had less pathological lesions across all models although only significantly so in the MIA and Saline model for modified Mankin (p<0.001 and p = 0.003, respectively), Pritzker (p<0.001 and p = 0.046, respectively), and Gerwin (p = 0.018 and p = 0.058, respectively) scoring systems. These findings demonstrate the MIA model produces profound, generalized cartilage degeneration within the weight-bearing surface of the joint with erosion of hyaline cartilage, mineralization of cartilage and bone, and destruction to tidemark integrity compared to surgical models (Fig 4). Furthermore, the damage induced by MIA extends further across the medial tibial plateaus and into the subchondral bone compared to the more localized lesions in the surgical models. The DMM model resulted in mild damage to the medial tibial plateaus of the ipsilateral joint 14 weeks post-OA induction. The Pritzker, modified Mankin, and Gerwin histological scores were higher in the DMM model after 14 weeks (9.83 ± 2.65, 5.21 ± 1.47, 2.33 ± 2.01, respectively) compared to the ACL+DMM model at 6 weeks (3.67 ± 3.50, 2.94 ± 2.22, 3.21 ± 1.83, respectively), though not significantly so. Importantly, histological scores in the two surgical OA groups were not appreciably different from the saline or untreated controls.

## Discussion

We found that the induction of OA using the MIA model caused significantly more cartilage destruction in the medial aspect of the stifle joint than the surgical models; however, both the ACL+DMM and MIA models showed persistent signs of gait impairment as assessed visually and objectively. Reflexive measures of sensitivity differed between the models, with the MIA model producing greater thermal hyposensitivity compared to other groups.

Reflexive testing remote from the affected joint has been the gold standard to assess ‘pain’ induced by OA, although such reflexive measures are measures of sensitivity, and not of nociception or pain. Here, overall, mechanical sensitivity was not significantly different between groups. However, the ACL+DMM surgery and MIA injection triggered significant short-lasting mechanical hypersensitivity, normalizing within 15 days. The overall lack of persistent mechanical hypersensitivity in this study is unusual; most studies report long-lasting mechanical sensitivity for at least 5 weeks [15] or up to 15 weeks post-OA induction [23]. A recent study reported a biphasic pattern of mechanical hypersensitivity, where sensitivity is greatest immediately (D1-5) and chronically (3–12 weeks) following surgical induction of OA (i.e., medial meniscal transection) [24]. The reason for the dissimilarity seen between our study and others is unclear; however, may be related to different experimental protocols (e.g., models, assays), study design, or environmental factors. Additionally and importantly, we did not select for animals that showed sensitivity as is performed in some studies [25–27], which likely explains some of the differences between our results and other reported work.

The MIA model induced significant thermal hypersensitivity compared to controls, and the ACL+DMM group suggesting differential effects of the MIA model on somatosensory systems. Similarly, the literature on thermal sensitivity in rodent OA models has shown differing effects across models. Generally, there is a strong association with thermal hypersensitivity in chemical models of OA [28], while in surgical models of OA no changes over time in thermal latencies are detected [23, 24, 29]. Previous reports found intraarticular injection of MIA in the stifle joint caused thermal hyperalgesia 28 days following injection [28]. Of note, our data, despite the attempts to control the contextual environment, were quite variable as can be seen with the saline group. However, as with the mechanical sensitivity testing, we did not select or remove any subjects. Varying effects of naturally occurring OA pain on thermal sensitivity (hyposensitivity and hypersensitivity) have also been found in canine [30] and human OA patients [31].

Limitations of reflexive tests include the varying and unpredictable effects of the experimenter, environment, and exact experiment conditions. However, another important limitation of reflex measures is that they are measures of changes in sensitivity, and do not reflect the aversive nature of pain (the affective components) [32]. Measures of more complex behaviors, including the ability to perform activities, are likely better measures of the aversive nature of pain. Similarly, measures of limb use in relation to joint disease likely more closely reflect pain itself.

Visual lameness was observed over a 6-week period, with persistently worse scores in the ACL+DMM group compared to the MIA group. Interestingly, the degree of lameness did not correlate with the severity of joint damage, as the MIA group had higher histological scores than ACL+DMM group. This underscores the poor relationship between measurable disease and pain seen in clinical cases [33]. As discussed later, the objective measures of limb use did not show such pronounced changes. Subjective lameness assessments were evaluated at the cage-side level without manipulation of the animal, therefore, minimizes the influencing factor of the experimenter presence. Additionally, the evaluation was done “at rest,” unlike the objective assessments of limb use. Although animals were acclimated to handling, objective clinical assays required the animals to be removed from home cage and repeatedly experience physical contact, potentially causing unintentional stress to the animals. The presence of humans or animals can impact pain behavior responses resulting in the animal masking their pain due to a protective response to avoid looking like prey [34], socialization [34], and/or stress-induced analgesia [35]. Similar findings are described in humans, where pain can either be heighted or reduced depending on the social environment [36]. Given our results, we believe, non-invasive visual evaluation of lameness may be advantageous and should be explored further, perhaps using remote monitoring and automated video analysis using a machine learning approach. It is important to note that there are no validated subjective assessments (indeed, none have been validated in higher-order animals such as dogs or cats either), but the assessments were done by investigators blind to group allocation. One potential reason for the higher lameness scores at rest in the ACL+DMM group may be the transection of the cruciate ligament, which introduces a degree of stifle instability, but it is unclear why these higher subjective scores were not reflected in the objective measurements.

Patients with stifle OA have an increase in biomechanical adduction therefore have higher mechanical loads on the medial compartment of stifle, which significantly contributes to OA progression [37]. Similar characteristics may occur with joint instability caused by the ACL+DMM surgical procedure. Although biomechanics were not directly measured, we evaluated functional limb use during ambulation and while standing. Gait was significantly altered in both OA-induction models compared to controls, characterized by: reduction in print length, print area, and stance and longer swing phase of the ipsilateral hindlimb. Additionally, static weight-bearing on ipsilateral hindlimb was substantially reduced in both OA groups over the first 28 days. Significant dynamic and static limb disability was shorter-lasting with peak dysfunction detected the day after OA-induction, comparable to other studies [24, 38, 39]. The similar results in both OA models suggests that pain associated with limb use is similar for both models. Similar changes to gait and weight-bearing have been found in canine [40, 41] and humans with hip or stifle OA [37]. Of note, though, is the fact that the degree of change in objective measures was overall relatively minor, begging the question as to whether such CatWalk measured parameters are a useful outcome measure in these models. Of course, another explanation for the overall relatively small changes from baseline at later time points is that these OA models are not associated with much impairment in limb use.

Six weeks following intraarticular injection of MIA, severe chondrocyte death, erosion of the hyaline cartilage, and remodeling of the subchondral bone was observed, similar to other studies [16]. Joint degeneration in the MIA model was significantly worse than the ACL+DMM model, assessed at the same time point, and also worse than the DMM model, evaluated 14 weeks following OA-induction. The ACL+DMM model produced minimal pathological changes that were not different from controls, which is most likely attributed to a relatively short period following OA-induction. Predominant lesions in the ACL+DMM model included superficial surface discontinuity and occasional cell proliferation. Other investigators using surgical models of OA have described similar severity of lesions at 4 and 6 weeks, but substantial cartilage degeneration (similar to what was found here in the MIA model) at later time points (e.g., 12 weeks) [24, 42, 43]. In studies evaluating the mouse DMM model, histological signs of OA have been detected as early as 2 weeks, with mild-to-moderate OA at 4 weeks, moderate-to-severe OA at 8 weeks, and progressively more severe OA at 16 weeks [17, 44]. Knowledge regarding histological changes associated with the rat DMM model is more limited. However, Choi et al. recently found more severe OA at 6 weeks, with similar OARSI scores 6 through 12 weeks [45]. Most investigators assess OA findings between 4 to 8 weeks in the rat DMM model [13, 46–48].

Interestingly, compared to mouse studies, there is a paucity of information on the progression of OA in the rat ACL+DMM and DMM models, which may be because minimal lesions occur and negative findings are not reported. These findings are in agreement with our hypothesis that the surgical OA models would have a slower progression of OA damage due to the localized damage caused by joint instability following the surgical manipulation. Significant degenerative lesions may have been detected in the ACL+DMM model if evaluated at later time points post-induction, as done in the DMM model. We presume that histological alterations would presumably be worse than the DMM if the same late time points were compared due to the combination of transection of the ACL and the DMM procedures. Future work should include later time points in the surgical OA models, directly compare surgical models at the same timepoints, and fully characterize the timeline of progression of both behavioral indicators of OA-associated pain, and histological progression of OA lesions in rat surgical OA models.

Differences in OA severity can be explained by the mechanisms associated with the specific OA model. Joint instability, caused by transection of the ACL and/or DMM, alters the biomechanical load on the weight-bearing surfaces of the joint (e.g., medial tibial plateau) because of anatomical displacement. Work in mice indicates that the progression of OA in surgical models is dependent on the severity of joint instability imposed [42]; work in the rat has shown that OA lesions may be exacerbated by increased weight-bearing [49]. In humans, several kinematic characteristics are known risk factors for stifle OA progression during gait [50]. In contrast, intraarticular injection of MIA inhibits chondrocyte glycolysis resulting in generalized chondrocyte damage and death, subchondral bone necrosis, and inflammation [51]. In the MIA model, cartilage degeneration is independent of instability and is generated via chondrocyte destruction.

Histology may not be representative of functional disability and pain in the patient. We found a disconnect between pathological severity of OA and clinical pain impairments. The surgical ACL+DMM model showed persistent signs of dysfunction across the functional measures but had mild-to-moderate histological features of OA. Whereas animals in the MIA group had moderate-to-severe histological joint damage but similar pain behaviors. These findings are in alignment with other experimental [24] and human OA studies [33]. In clinical studies, pathological severity is typically scored radiographically. High grade radiographic evidence of knee OA in humans has shown variable strengths of relationship with pain severity, with some finding strong correlations [52, 53] while others showing poor correlations [54, 55]. It is unclear why a disparity exists between pain severity and joint pathology. Emerging data indicate OA is no longer predominantly a disease of the cartilage, but other complex factors, such as mechanistic involvement, can lead to progressive joint destruction and severe chronic pain [56]. The progression of damage to the joint in OA has been associated with central sensitization [33, 57]. Pain-associated behavioral responses are dependent on mechanisms of peripheral and central sensitization driven by sensitized nociceptive primary afferents and plasticity and reorganization of cortical circuitry, where pain is perceived [58]. Further work should be performed in these rat OA models to elucidate the mechanisms responsible for pain in the different models–differing mechanism of pain in the different models may make one model preferred over the other depending on the experimental question.

Despite the widespread use of OA models in the rat, no work has been performed to define the relative predictive utility of the various models–that is, define which models better predict therapeutic utility in humans. This is an important consideration for future research.

This was a pilot study, and as such, has limitations. First, varying numbers of animals were used for each experimental group with variable distribution of sexes because data were combined from different contemporaneous experiments. Therefore, sex differences could not be determined. However, the experimenters, testing environment, and protocols remained consist for all data evaluated. Secondly, clinical pain behaviors were not recorded in the DMM model, limiting comparison and discussion between this model and the other OA models. Another limitation pertains to the control groups–we did not include a sham surgery group, only an intra-articular saline administration control, and untreated controls for histological comparisons. While we felt that this approach was appropriate for this pilot work, future work focusing on the time course of pain behaviors and histological changes in surgical models should include sham surgery controls as well as untreated, age-matched controls.

In summary, our histological analysis and clinical evaluation of pain for 6 weeks following ACL+DMM or MIA OA-induction demonstrated similarities to human stifle OA, including cartilage destruction, mechanical hypersensitivity, and reduced dynamic and static limb use. However, there appeared to be little difference between the OA models in terms of measures of pain (limb use). The MIA model demonstrated thermal sensitivity, as has been described previously, but the value of this in translational research is yet to be determined. Histological changes were more profound in the MIA model, potentially making it more suitable for quickly screening novel therapeutics targeted towards mitigating or reversing histological changes. The histological changes in the surgical OA models were limited, and not different from controls, suggesting that an adaptation of the methods of inducing OA may be required to increase the severity of OA. Future studies should repeat this work but extend the measures to include complex behavioral assays of pain, and biochemical, molecular, and imaging analysis of both sensory system and joint tissue changes in order to fully characterize the models. Future studies should evaluate these surgical OA models for more chronic periods to further evaluate longitudinal changes in histological degeneration and behavior. This will allow for more informed decisions regarding the applicability of a particular model to an investigative question. In this study, we found that the chemical OA model (MIA) produced profound histopathological OA lesions in relatively shorter periods of time compared to surgical models (ACL+DMM, DMM), despite similar gait impairment outcome measures.

## Supporting information

S1 Fig Comparison of quantitative sensory testing following OA induction. Bar plot of mechanical paw withdrawal threshold (a) overall and (b) change from baseline over time. Thermal paw withdrawal latency graphs show (c) overall changes and (d) changes from baseline over time. Data are expressed as the mean ± SEM. *p<0.05, **p<0.01, ***p<0.001 compared to baseline.
(TIF)

S2 Fig Changes from baseline in ipsilateral limb use following OA induction over time. Graphs of (a-e) dynamic gait and (f) static weight distribution on ipsilateral hindlimb following OA-induction show change from baseline over time for (a) stance phase, (b) print length, (c) print area, (d) swing phase, (e) time to max paw-floor contact, and (f) weight distribution. Data are expressed as the mean ± SEM. *p<0.05, **p<0.01, ***p<0.001 compared to baseline; ^p<0.05, ^^p<0.01, ^^^p<0.001 compared to saline; ^ϕ^p<0.05, ^ϕϕ^p<0.01, ^ϕϕϕ^p<0.001 ACL+DMM vs MIA.
(TIF)
