# Supplementary figures and images for: Pilot comparison of outcome measures across chemical and surgical experimental models of chronic osteoarthritis in the rat (Rattus norvegicus)
Source: PLoS One. 2022 Nov 21;17(11):e0277943. doi: 10.1371/journal.pone.0277943 (PMC9678322; doi:10.1371/journal.pone.0277943)

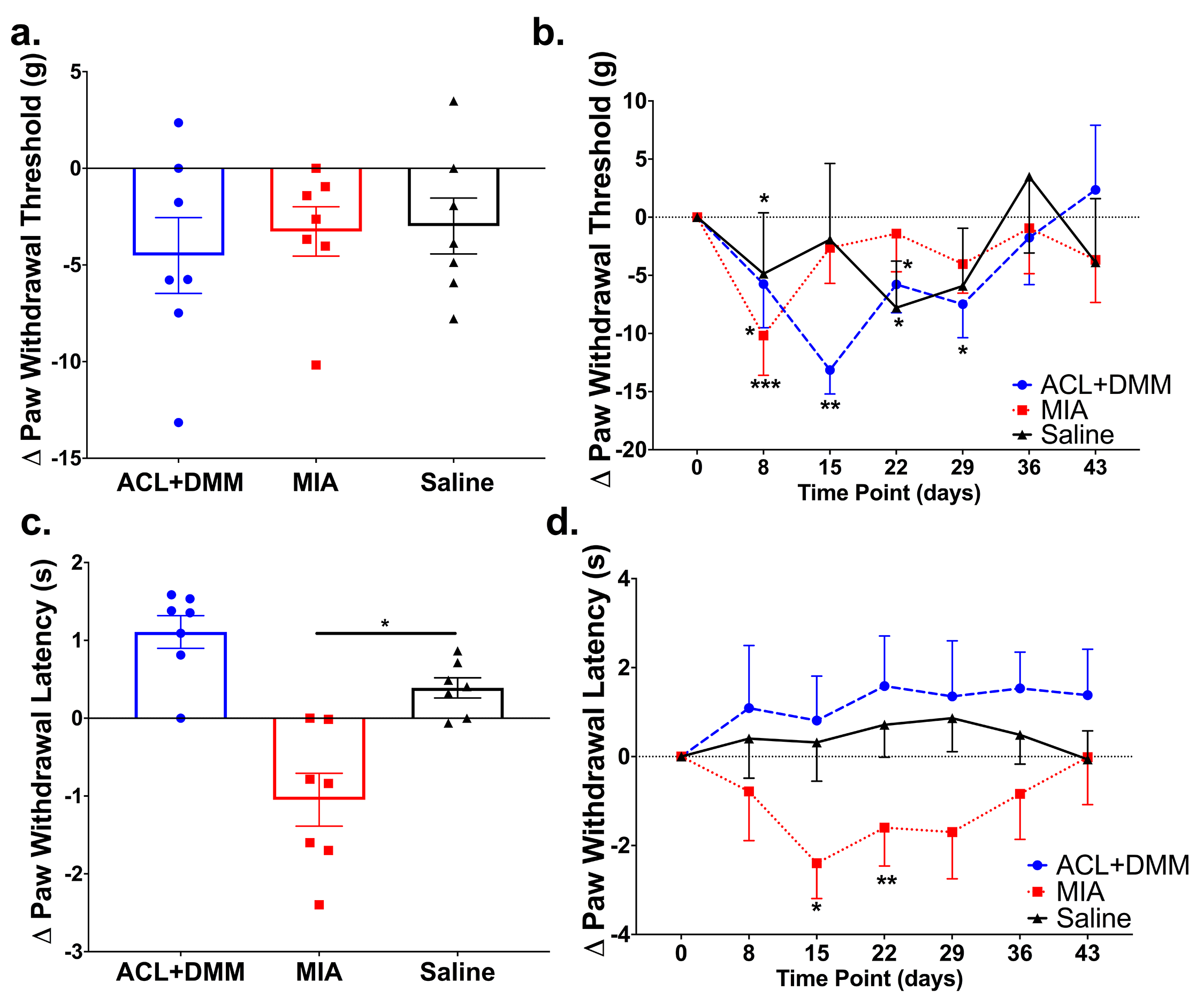

Supplement: S1 Fig — Bar plot of mechanical paw withdrawal threshold (a) overall and (b) change from baseline over time. Thermal paw withdrawal latency graphs show (c) overall changes and (d) changes from baseline over time. Data are expressed as the mean ± SEM. *p<0.05, **p<0.01, ***p<0.001 compared to baseline. (TIF) [file pone.0277943.s001.tif]

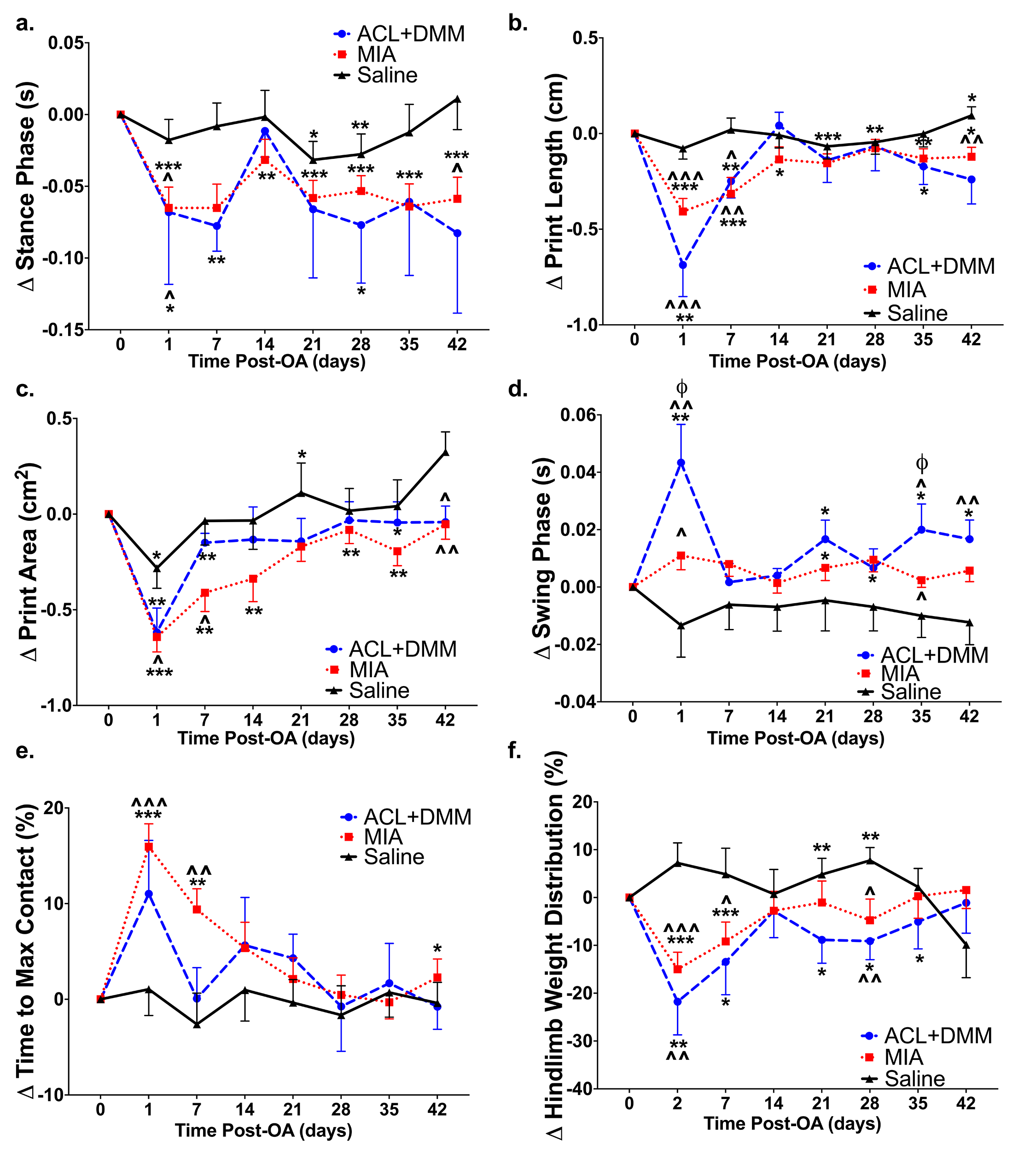

Supplement: S2 Fig — Graphs of (a-e) dynamic gait and (f) static weight distribution on ipsilateral hindlimb following OA-induction show change from baseline over time for (a) stance phase, (b) print length, (c) print area, (d) swing phase, (e) time to max paw-floor contact, and (f) weight distribution. Data are expressed as the mean ± SEM. *p<0.05, **p<0.01, ***p<0.001 compared to baseline; ^p<0.05, ^^p<0.01, ^^^p<0.001 compared to saline; ϕp<0.05, ϕϕp<0.01, ϕϕϕp<0.001 ACL+DMM vs MIA. (TIF) [file pone.0277943.s002.tif]
